# Charge and Spin Dynamics and Enantioselectivity in Chiral Molecules

**DOI:** 10.1021/acs.jpclett.1c03925

**Published:** 2022-01-24

**Authors:** J. Fransson

**Affiliations:** Department of Physics and Astronomy, Uppsala University, Box 516, 751 21 Uppsala, Sweden

## Abstract

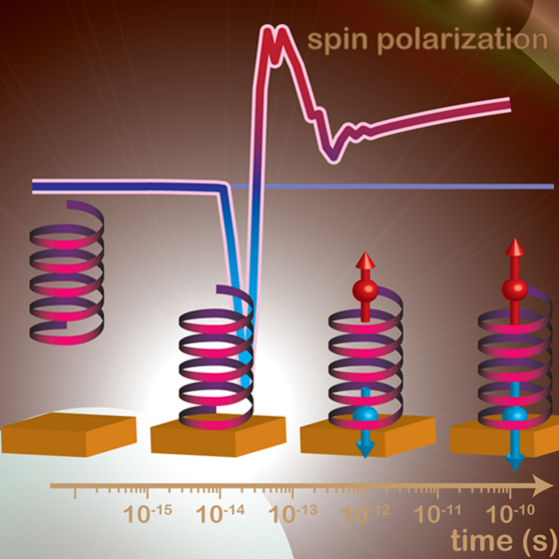

Charge and spin dynamics are addressed in chiral molecules immediately after their instantaneous coupling to an external metallic reservoir. This work describes how a spin polarization is induced in the chiral structure as a response to the charge dynamics. The dynamics indicate that chiral induced spin selectivity is an excited state phenomenon that in the transient regime can be partly captured using a simplistic single-particle description but in the stationary limit definitively shows that electron correlations, e.g., electron–vibration interactions, crucially contribute to sustain an intrinsic spin anisotropy that can lead to a nonvanishing spin selectivity. The dynamics, moreover, provide insight into enantiomer separation, due to different acquired spin polarizations.

For more than two decades, we have learned that electron spin selective processes are intimately associated with chirality.^1,2^ The effect can be partly understood as emerging from a combination of structural chirality, spin–orbit interactions, and non-equilibrium conditions.^3−28^ Despite the existence of non-equilibrium conditions, which define the measurements, for instance, light exposure,^1,2,31−36^ local probing techniques,^37−40^ transport,^38,41,42^ and different types of Hall measurements,^32,33,43−45^ many theoretical accounts of the effect are based on the transmission properties of chiral molecules embedded in a given environment.^3−8,10−13,15−17,19,20^ While the transmission pertains to the linear response regime, hence, the ground state properties of the molecule, it is also often typically the result of a single-particle description that under stationary conditions cannot account for the excited state properties that underlie spin selectivity in chiral molecules.

Recent theoretical developments very clearly point to the need to consider chiral induced spin selectivity from an excited state point of view,^18,21−23,25−30^ stressing the vital role of electronic correlations. In this context, excited states refer to, e.g., virtual excitations, temporal fluctuation-induced electron dispersion within the spectrum, and thermally induced vibrational excitations of the molecule that couple to the electron and, hence, strongly modify the electronic structure. There might, however, be other sources of excitation of the molecule. It was shown that, e.g., Coulomb^18,25,29^ and electron–vibration^21,23,24,30^ interactions, as well as polarons,^22^ photoexcitations,^26^ time dependence,^27^ and dissipation,^28^ generate the exchange necessary to create measurable effects regarding chiral induced spin selectivity.

In this context, an obvious question is how a spin polarization can be generated and stabilized in a molecular structure that is in a closed shell configuration when isolated from the surrounding environment. Recent experiments demonstrate that a measurable spin polarization can be obtained whenever chiral molecules are interfaced with metallic surfaces.^43,46−48^ Through the anomalous Hall effect, chiral molecules were, for instance, shown to control the magnetism in thin Co layers^43,46^ and enantiomer separation on nonmagnetic metals,^47^ whereas Yu–Shiba–Rusinov states^49−51^ were observed in the vicinity of chiral molecules on the surface of superconducting NbSe_2_.^48^ Related to these observations are also the results showing strongly enantiomer-dependent binding energies on ferromagnetic metals.^52−57^ Theoretically, enantiomer separation was addressed in refs (29) and (30) for molecules in contact with ferromagnetic metal, based on descriptions in which the effective electronic exchange plays a crucial role in the magnetic response. On the contrary, while excited states appear to be crucial, the question of how spin polarization emerges in chiral molecules when they are in contact with a metal remains open.

This Letter shows that a finite spin polarization is dynamically generated in chiral molecules as a response to the charge dynamics when interfaced with a metal. The dynamics indicate that chiral induced spin selectivity is an excited state phenomenon that in the transient regime can be partly captured using a simplistic single-particle description, while such a description is not sufficient in the stationary limit. The latter statement is founded on the idea that the spin polarization eventually vanishes, which, in turn, implies that any mechanism that can sustain an immanent spin anisotropy must account for excited state properties, e.g., electron–electron or electron–vibration interactions. In the subsequent discussion, it is, therefore, shown that, upon addition of electron–vibration interactions, the transient spin fluctuations are stabilized and developed into a finite spin polarization as the stationary limit is approached. Finally, when the different enantiomers are interfaced on a ferromagnet, the results presented herein provide fundamental clues for the development of a comprehensive picture of enantiomer separation.

The discussion presented here is based on simulations of idealized chiral models of realistic, e.g., α-helix, oligopeptides, polyalanines, and helicene. In this context, it is important that while the specific details of the molecules used in the experiments strongly vary, the salient properties such as chirality, spin–orbit interaction, and interface to a metal are captured within this model. The model was previously proposed to illustrate the importance of electronic correlations originating from Coulomb^18^ and electron–vibration^23,30^ interactions. Here, such mechanisms are first excluded for the sake of highlighting the dynamics of excited states as a fundamental component in the charge redistribution and accompanying spin polarization immediately after the molecule is interfaced with the metal. Then, in the subsequent discussion, I show that electron–vibration interactions stabilize a nonvanishing spin polarization also in the stationary limit, hence emphasizing that the phenomenon of chiral induced spin selectivity is an excited state effect.

The drawings in Figure 1 illustrates the electronic processes within the molecule before and after contact with the metal is made. Transitions (arrows) between the main electronic states (bold), the vibrationally induced states (faint and dotted), or the main and vibrationally induced states are not allowed (crosses) by orthogonality of the electronic spectrum. By contrast, the presence of the metal breaks up the orthogonality of the spectrum, which allows all transitions that were forbidden before contact.

**Figure 1:**
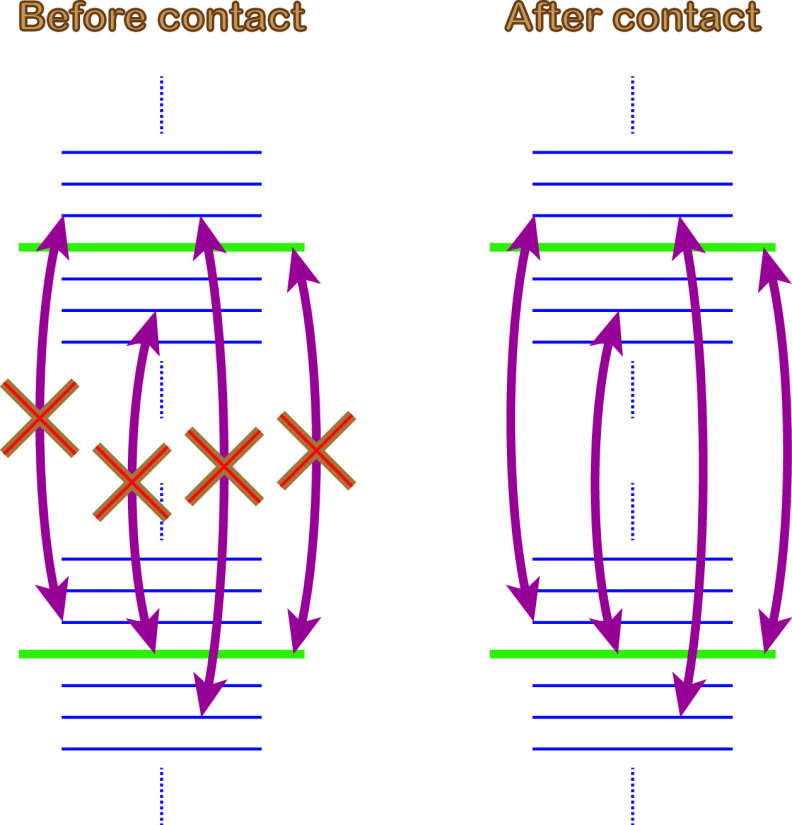
Schematic drawing of the electronic processes in the chiral molecule before and after contact with the metal. Bold (faint and dotted) lines represent the main (vibrationally induced) electronic levels, and arrows signify a few possible transitions between the states that before contact are not allowed by orthogonality. On the contrary, these are allowed after contact with the metal is made because the presence of the metal breaks up the orthogonality between these states.

The simulations are based on a model of a chiral structure comprising a set of  nuclear coordinates **r**_*m*_ = (*a* cos φ_*m*_, *a* sin φ_*m*_, *c*_*m*_), , and *c*_*m*_ = *cφ*_*m*_/2π, where *a* and *c* define the radius and length, respectively, of the helix of *M* laps and *N* nuclei per lap. The molecule is described by the single-electron levels , representing the energy levels at **r**_*m*_, associated with the electron creation and annihilation spinors ψ_*m*_^†^ and ψ_*m*_, respectively. For equivalent sites, assuming that ε_*m*_ = ε_0_ for all *m* is justified. Nearest neighbors interact via elastic and inelastic hopping with rates *t*_0_ and *t*_1_, respectively, while next-nearest neighbors interact via the effective static and nonstatic spin–orbit interaction with rates λ_0_ and λ_1_, through processes of the type *iψ*_*m*_^†^**v**_*m*_^(*s*)^·**σ**ψ_*m*+2*s*_, where *s* = ±1, where **v**_*m*_^(*s*)^ = **d̂**_*m*+*s*_·**d̂**_*m*+2*s*_, which defines the chirality in terms of the unit vectors **d̂**_*m*+*s*_ = (**r**_*m*_ – **r**_*m*+*s*_)/|**r**_*m*_ – **r**_*m*+*s*_|, such that different enantiomers are represented by the sign (±) of the chirality. Coupling the chirality with the spin–orbit interaction mechanism is justified because the geometry of the structure inevitably can be related to an intrinsic electric field. The notations σ^0^ and **σ** refer to the identity and vector of Pauli matrices, respectively. The nuclear or molecular vibrations are captured in the coherent vibrational mode ω_0_, which is created and annihilated by the phonon operators *b*^†^ and *b*, respectively. A Hamiltonian model can be written 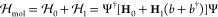
where  and
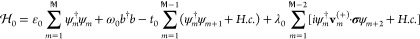
1a
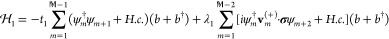
1bThe properties of the metal to which the molecule is connected are captured by the parameter **Γ** = Γ(σ^0^ + *pσ*^*z*^)/2, representing the coupling between nuclear site 1 and the metal. Here, Γ = 2π∑_**k**σ_|*v*_**k**σ_|^2^ρ_σ_(ε_**k**_) accounts for the spin-dependent hybridization *v*_**k**σ_ and spin density ρ_σ_(ε_**k**_) of the electrons in the metal, whereas |*p*| ≤ 1 denotes the effective spin polarization of the coupling.

The time evolution of the electronic structure of the molecule can be related to the time-dependent Green function **G**_*mn*_(*t*, *t*′) = (−*i*)⟨*Tψ*_*m*_(*t*)ψ_*n*_^†^(*t*′)⟩ through, e.g., the charge ⟨*n*_*m*_(*t*)⟩ = (−*i*)sp**G**_*mm*_^<^(*t*, *t*) and spin moment ⟨**s**(*t*)⟩ = (−*i*)sp**σG**_*mm*_^<^(*t*, *t*)/2, where **G**^</>^(*t*, *t*′) is proportional to the density of occupied/unoccupied electron states. Here, sp denotes the trace over spin = ^1^/_2_ space.

As the main interest here lies on the time dependence of the molecular properties immediately after the time *T*_0_ when the molecule is interfaced with the metal, the interaction with the metal is treated with a time-dependent hybridization *v*_**k**σ_(*t*) = *v*_**k**σ_θ(*t* – *T*_0_), where θ(*t*) is the Heaviside step function. In light of this time-dependent interaction, the equation of motion for the static (**H**_1_ = 0) Green function can be written as the Dyson equation
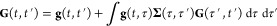
2a
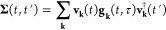
2bThe bare Green functions **g**(*t*, *t*′) = (−*i*)*T*e^–*i***H**_0_(*t*–*t*′)^ and **g**_**k**_(*t*, *t*′) = (−*i*)*T*σ^0^e^–*iε*_**k**_(*t*, *t*′)^ carry trivial time dependencies, and alluding to the wide band limit for the electronic band in the metal,^58^ the lesser self-energy can be simplified into
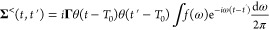
3where *f*(ω) is the Fermi–Dirac distribution function defined at the chemical potential μ of the metal. Because of this simplification, and the relation **G**^<^ = **G**^r^**Σ**^<^**G**^a^, where **G**^r^ and **G**^a^ are the retarded and advanced Green functions, respectively, the dressed lesser and retarded/advanced Green functions for the molecule become
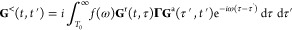
4a
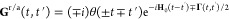
4bwhere the expression in eq 4a is valid for *t*, *t*′ > *T*_0_, whereas **Γ**(*t*, *t*′) = **Γ**∫_*t*′_^*t*^θ(*s* – *T*_0_) d*s*.

Effects from inelastic scattering are included by repeating eq 2a, defining a dressed Green function  and self-energy **Σ**_vib_(*t*, *t*′) = *i***H**_1_**g**(*t*, *t*′)*d*(*t*, *t*′)**H**_1_, where *d*(*t*, *t*′) = 2*T* sin ω_0_(*t* – *t*′) is the propagator for the nuclear vibrations. However, because the aim is to provide a simple description of the charge and spin dynamics and a mechanism that eventually leads to a nonvanishing spin-polarized stationary state, as discussed in ref (30), the full Dyson equation for  is reduced to the Markovian approximation, in which the self-energy becomes time-independent. In this form, the resulting retarded Green function can be written as
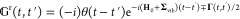
5in which expression **Σ**_vib_ = **H**_1_**LH**_1_, where **L** is a diagonal matrix where the entries are defined by the electron–phonon loop
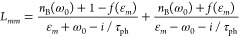
6and *n*_B_(ω) is the Bose–Einstein distribution function, whereas τ_ph_ defines an intrinsic vibrational lifetime. This lifetime arises from vibration–vibration and vibration–electron interactions and reflects the conditions of the environment.

In a previous discussion, it was shown that the molecular structure given in **H**_0_, eq 1a, carries necessary components that lift the spin degeneracy, arising from a confluence of the spin–orbit interactions and the chiral geometry.^18^ However, the structure has to include at least four nuclear sites and be under non-equilibrium conditions, of which the latter was previously considered to be provided by an external voltage bias. The lack of a coupling between the internal and dissipative degrees of freedom does, nonetheless, not allow the structure to maintain a stationary spin polarization when held in equilibrium with one end attached to a metal (see ref (30)). Such a mechanism is provided by the coupling between electronic and vibrational degrees of freedom in **H**_1_ (eq 1b), where the spin-independent and -dependent components broaden and introduce a spin exchange, respectively, into the spectrum. Hence, it is shown that the elastic spectrum provided through **H**_0_ captures only strictly non-equilibrium properties, while the stationary properties emerge from **H**_1_. The discussion stresses that while electron correlations are necessary for an adequate description, the single-electron picture provides crucial insight into the emergence of the spin symmetry breaking.

Within the given model (eq 1), the magnetic moment of the molecule vanishes whenever the molecule is isolated from the metallic environment. When the molecule is attached to the metal, the molecule is set into a strongly non-equilibrium state where electrons are passing through the interface between the molecule and metal, rendering a redistribution of charge in the molecule. These processes are captured very well already at the single-electron level (**H**_1_ = 0) (see Figure 2a,e), where a spatially resolved time evolution of the molecular charge, ⟨*n*_*m*_⟩, is shown for a (6 × 8 sites, ε_*m*_ = ε_0_, for all values of *m*) chiral molecule with (a) positive and (e) negative chirality immediately after the instantaneous attachment of the molecule to a normal metal, with the parameters summarized in Table 1. The plots show that the time evolutions of the charge distributions of two enantiomers are the same. The plots also show the strong fluctuations of the charge due to the abrupt changes in the environment. For instance, immediately after contact with the metal, the total molecular charge is reduced and restored shortly thereafter, accompanied by a strongly fluctuating charge polarization that eventually wanes (see the inset of Figure 2a) as a result of electrons flowing between the molecule and metal.

**Table 1:** Model Parameters Used for the Simulations in Units of *t*_0_ = 1 eV

	*t*_1_	λ_0_	λ_1_	ε_0_ – μ	ω_0_	Γ
static molecule	0	10^–3^	0	–4	–	0.01
vibrating molecule	0.04	10^–3^	10^–4^	–4	10^–4^	0.01

**Figure 2:**
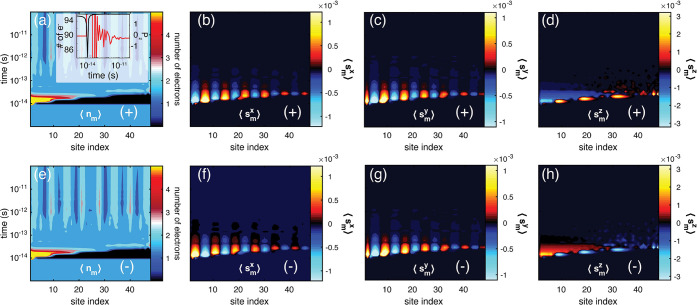
Single-electron picture of the charge and spin dynamics in a chiral molecule (6 × 8) before and after making instantaneous contact with a metal at time *T*_0_ = 10 fs. (a and e) The spatially resolved charge ⟨*n*_*m*_⟩ and spin projections (b and f) ⟨*s*_*m*_^*x*^⟩, (c and g) ⟨*s*_*m*_^*y*^⟩, and (d and h) ⟨*s*_*m*_^*z*^⟩ are simulated for positive (+) (a–d) and negative (−) (e–h) helicity. The inset of panel a shows the total number of electrons (black) and charge polarization (red) in the molecule as a function of time. The following parameters were used: *a* = 5 Å, *c* = 235 Å, ε_0_ – μ = −4, λ_0_ = 1/1000, and Γ = 1/100, in units of *t*_0_ = 1 eV, simulated at *T* = 300 K.

The charge motion in the chiral molecule generates an accompanied spin polarization that evolves in both time and space (see Figure 2b–d,f–h), showing the projections of ⟨**s**_*m*_⟩ for the two enantiomers. The plots illustrate that there is a finite time frame during which the spin polarization is significant, after which it wanes and eventually vanishes (between 0.1 and 1 ps after contact).

With regard to spin polarization, there are two distinctive features that are clearly illustrated in Figure 2. First, the transverse projections, ⟨*s*_*m*_^*x*,*y*^⟩, indicate antiferromagnetic spin configurations resulting in no, or little, net moment perpendicular to the length direction of the molecule. This is in sharp contrast to the longitudinal projection ⟨*s*_*m*_^*z*^⟩, indicating a more uniform distribution in the structure. Second, the two enantiomers acquire opposite spin polarizations such that the spin moments ⟨**s**_*m*_⟩_±_ = (⟨*s*_*m*_^*x*^⟩_±_, ⟨*s*_*m*_^*y*^⟩_±_, ⟨*s*_*m*_^*z*^⟩_±_) in the ± enantiomers, respectively, are related by rotating the spins around the *y*-projection, that is, (⟨*s*_*m*_^*x*^⟩_–_, ⟨*s*_*m*_^*y*^⟩_–_, ⟨*s*_*m*_^*z*^⟩_–_) = (−⟨*s*_*m*_^*x*^⟩_+_, ⟨*s*_*m*_^*y*^⟩_+_, −⟨*s*_*m*_^*z*^⟩_+_).

Inclusion of molecular vibrations changes the qualitative aspects of the dynamics (see Figure 3), showing the spatially resolved time evolution of the molecular charge and spin from simulations with vibrating chiral molecules. First, one can notice that the vibrationally assisted spatial evolution is qualitatively similar to the evolution in the purely static molecule. However, while the charge in the static molecule returns to a nearly homogeneous distribution in the stationary regime (Figure 2a,e), in the vibrating molecule the charge distribution remains inhomogeneous, acquiring a nonvanishing charge polarization also when the system dynamics is slowed, approaching the stationary limit (see panels a and e of Figure 3 and inset of panel a). Second, one can notice that the spin polarizations that develop shortly after contact with the metal also remain upon approaching the stationary state (Figure 3b–d,f–h). While the transverse projections are configured antiferromagnetically within the molecule (Figure 3b,c,f,g), the longitudinal projection converges toward a spin-polarized state with opposite polarizations at the two ends of the molecule (Figure 3d,h). The state that is eventually reached for the molecule in the regime with slow dynamics corroborates the results obtained in the stationary regime,^30^ albeit the two approximations used are not exactly equivalent. The consistency between the results, nonetheless, suggests that the dissipative component provided through the molecular vibrations should be realistic and sound.

**Figure 3:**
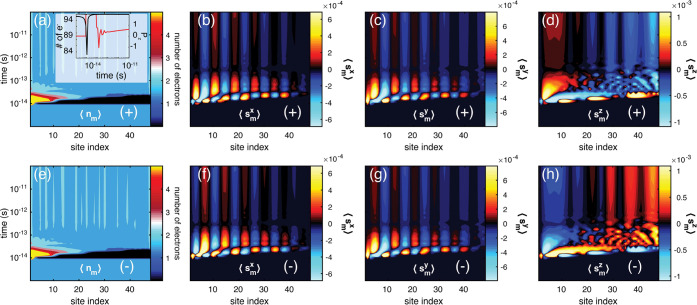
Charge and spin dynamics in a vibrating chiral molecule (6 × 8) before and after making instantaneous contact with a metal at time *T*_0_ = 10 fs. (a and e) The spatially resolved charge ⟨*n*_*m*_⟩ and spin projections (b and f) ⟨*s*_*m*_^*x*^⟩, (c and g) ⟨*s*_*m*_^*y*^⟩, and (d and h) ⟨*s*_*m*_^*z*^⟩ are simulated for positive (+) (a–d) and negative (−) (e–h) helicity. The inset of panel a shows the total number of electrons (black) and charge polarization (red) in the molecule as a function of time. The following parameters were used: *t*_1_ = 1/25, λ_1_ = 1/10000, ω_0_ = 1/10000, and 1/τ_ph_ = 1/100, in units of *t*_0_ = 1 eV. The other parameters are as described in Figure 2.

While the two enantiomers are expected to acquire opposite spin polarization when in contact with the metal, in the next step it will be shown that this feature also opens up for enantiomer separation. In experiments, this can be done by contacting the enantiomers on a ferromagnetic surface and measuring, e.g., the adsorption rate^52^ or the force required to pull the molecule off of the surface.^55^ Here, the enantiomer selectivity is studied through the magnetic properties of the composite system comprising both the molecule and the metal.

Using spin polarization as a tool for enantiomer separation was also considered in recent theoretical studies.^29,59^ However, quantifying the enantiomer specific properties in terms of, e.g., the transmission, which is essentially not a measurable quantity, introduces ambiguities into how to comprehend the results. The reason is that measurable quantities, such as the magnetic moment and charge current, are integrated over many degrees of freedom, in particular the energy dependencies of the involved mechanisms. Therefore, whether the detailed spectral properties are meaningful in a context in which this cannot be resolved is questionable.

Hence, here the enantiomer specific properties are investigated in terms of the mean spin polarization *M*^*z*^ = 2∑_*m*_(⟨*z*⟩ – *z*_*m*_)⟨*s*_*m*_^*z*^⟩/*c*, where  and the origin is located at the molecule–metal interface, which defines a measure using which it is possible to determine whether there is any difference in the magnetic moments between the two enantiomers. As such, a positive (negative) spin polarization can be understood as spin ↑ (↓) accumulating toward the interface with the metal accompanied by a spin ↑ (↓) depletion toward the free end of the molecule. The advantage with this measure is that it directly connects to integrated measurable quantities such as the total magnetic moment because it provides a relation between the local magnetic moments ⟨*s*_*m*_^*z*^⟩ and the overall structure.

The mean spin polarizations of static and vibrating molecules when in contact with a metal are shown in Figure 4. Bold (faint) lines in panels a and b correspond to simulations of the molecule in contact with a ferromagnetic (normal) metal with *p* = 0.1 (*p* = 0), while the plots in panel c show the difference *ΔM*^*z*^ = *M*_+_^*z*^ – *M*_–_^*z*^, where *M*_±_^*z*^ denotes the spin polarization for the ± enantiomers, for the vibrating (bold) and static (faint) configurations. Although the quantitative details vary between the static and vibrating configurations, the overall conclusion that can be drawn is that the enantiomers acquire different mean spin polarizations, particularly in the transient regime. While the static configuration does not allow for a distinction between the enantiomers when approaching the stationary limit, the vibrating configurations acquire a finite stationary mean spin polarization. Of great importance for the chiral induced spin selectivity effect, however, is that the two enantiomers acquire different amplitudes of their spin polarizations also in the stationary limit.

**Figure 4:**
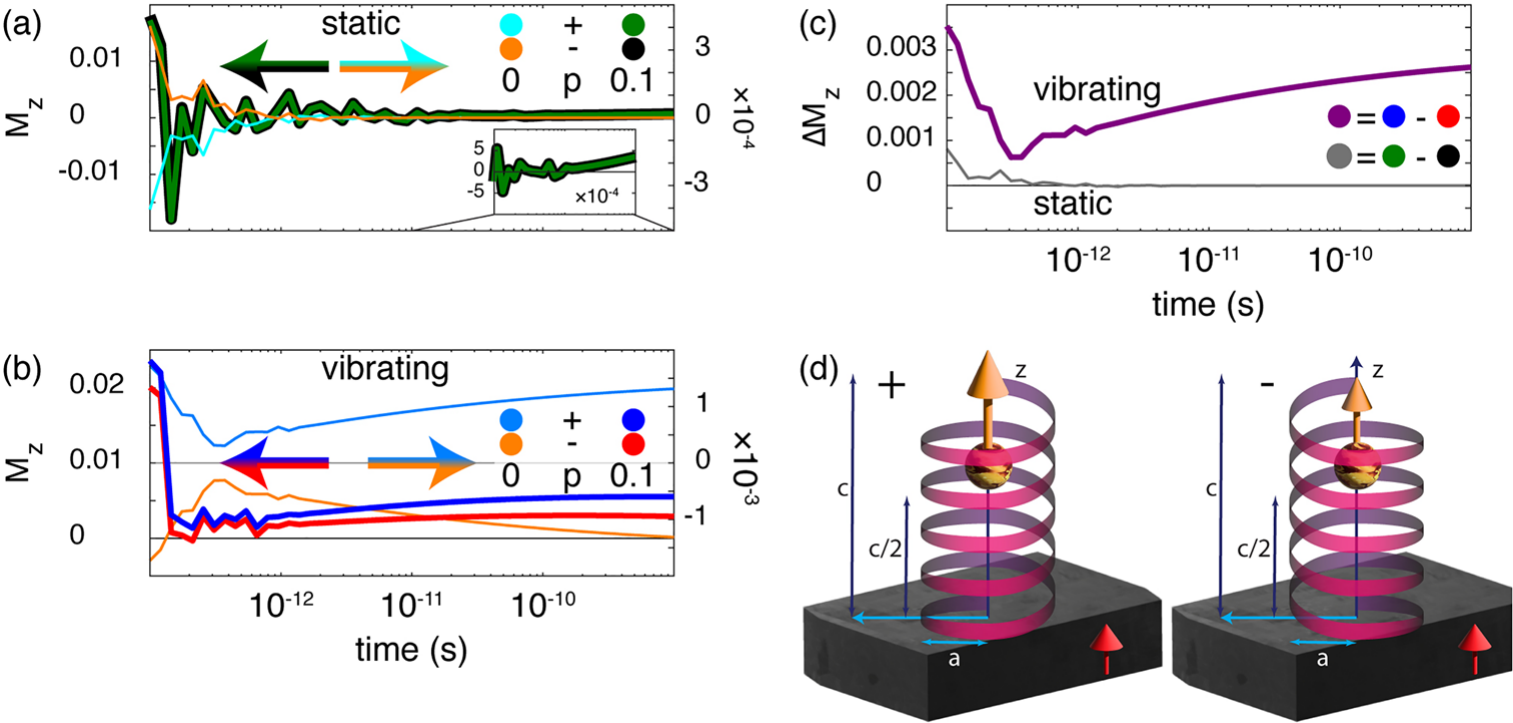
Time evolution of mean spin polarization *M*_±_^*z*^ for (a) a static molecule and (b) a vibrating molecule and (c) differences (*ΔM*_*z*_) between the enantiomers in the static and vibrating configurations. Bold (faint) lines in panels a and b represent the results in the presence of a ferromagnetic (normal) metal with *p* = 0.1 (*p* = 0) for the two enantiomers (±). In panel c, the bold (faint) line represents the vibrating (static) configurations. (d) Schematic illustration of the chiral molecule when attached to the metal, defining the origin at the molecule–metal interface. The drawings also illustrate the induced mean spin polarization immediately after the molecule contacts the ferromagnetic metal. The magnetic moment of the metal is indicated with the arrow. The mean spin polarization in the molecule with positive helicity is larger than that in the molecule with negative helicity. Other parameters are as described in Figure 3.

The results of the simulations can be interpreted as the schematic illustrations in Figure 4d, where the ball-arrow suggests the mean spin polarization of the + (left) and – (right) enantiomers when in contact with the ferromagnetic substrate. Hence, the chirality of the + (−) enantiomer cooperates (counteracts) with the ferromagnetism in the substrate, leading to an enhanced (reduced) mean spin polarization as the system approaches the stationary regime.

Here, one should stress that chiral induced spin selectivity does not originate from the emergence of a spin polarization in the molecule itself, but it is the unequal amplitudes of the spin polarizations of the enantiomers that form the basis for the phenomenon. This statement can be understood from the following discussion. The chiral induced spin selectivity effect is founded on the difference in the charge currents through a chiral molecule under different conditions. It can, for instance, be electrons photoexcited by circularly polarized light with opposite helicity such that the two photocurrents are different.^2^ While there is no question that the environment has a strong effect on the magnetic properties of the molecule, unless there is an immanent property of the molecule that responds differently when the light helicity is changed, there cannot be any change in the spin polarization of the photoelectric current. In this sense, the emergence of the spin polariztion is intimately related to the excited states of the molecule. In the transient regime, these excitations are made available by the dynamical changes of the electronic structure; however, when the stationary regime is approached, those excitations are no longer accessible in the static configuration considered here. That is, in the static configuration, there is no mechanism that allows for the transfer of electron density between states and, in particular, there is no mechanism that sustains an angular momentum transfer within the molecule. Nuclear or molecular vibrations, by contrast, facilitate transfer of both electron density and angular momentum between states or channels in the molecule, respectively. Moreover, at room temperature, there is a wide energy window available (∼26 meV) for electrons to transfer between states through thermal excitations, which is a reason for the effectiveness of the nuclear vibrations in this context.

The assumption of instantaneous attachment of the molecule to the metal is crude and may cause unrealistically large charge transfer within the molecule in the simulations. However, because the approximation can be regarded as a limiting process of any non-adiabatic attachment to the metal, it does provide the conceptual mechanism in this context.

In summary, spin polarization has been shown to be dynamically generated in chiral molecules upon them interfacing with a metal. The dynamics indicate that chiral induced spin selectivity is an excited state phenomenon that in the transient regime is sustained by the strong fluctuations imposed by the temporally changing conditions. When the stationary regime is approached, the system drives toward a ground state that may be spin-polarized; however, the existence of a nonvanishing spin polarization depends on whether the electronic density and angular momentum can be transferred between the states in the ground state. As I have shown here, nuclear vibrations do allow for such transfer and these results provide fundamental clues for the further development of a comprehensive theory for enantiomer selectivity.
